# Characterization of Venom and Oviduct Components of Parasitoid Wasp *Asobara japonica*

**DOI:** 10.1371/journal.pone.0160210

**Published:** 2016-07-28

**Authors:** Shunsuke Furihata, Takashi Matsumura, Makiko Hirata, Tetsuya Mizutani, Noriyo Nagata, Michiyo Kataoka, Yukie Katayama, Tsutomu Omatsu, Hitoshi Matsumoto, Yoichi Hayakawa

**Affiliations:** 1 Department of Applied Biological Sciences, Saga University, Saga 840–8502, Japan; 2 Research and Education Center for Prevention of Global Infectious Diseases of Animals, Tokyo University of Agriculture and Technology, Fuchu, Tokyo, 183–8509, Japan; 3 Department of Virology II, National Institute of Infectious Diseases, Tokyo, 280–0011, Japan; Institute of Plant Physiology and Ecology, CHINA

## Abstract

During natural parasitization, *Asobara japonica* wasps introduce lateral oviduct (LO) components into their *Drosophila* hosts soon after the venom injection to neutralize its strong toxicity; otherwise, the host will die. Although the orchestrated relationship between the venom and LO components necessary for successful parasitism has attracted the attention of many researchers in this field, the molecular natures of both factors remain ambiguous. We here showed that precipitation of the venom components by ultracentrifugation yielded a toxic fraction that was inactivated by ultraviolet light irradiation, boiling, and sonication, suggesting that it is a virus-like entity. Morphological observation of the precipitate after ultracentrifugation showed small spherical heterogeneous virus-like particles 20–40 nm in diameter. The venom’s detrimental effect on *D*. *melanogaster* larvae was not directly neutralized by the LO components but blocked by a hemolymphal neutralizing factor activated by the LO factor. Furthermore, we found that *A*. *japonica* venom and LO components acted similarly on the larvae of the common cutworm *Spodoptera litura*: the venom injection caused mortality but coinjection of the LO factor protected *S*. *litura* larvae from the venom’s toxicity. In contrast, *D*. *ficusphila* and *D*. *bipectinata*, which are closely related to *D*. *melanogaster* but non-habitual host species of *A*. *japonica*, were not negatively affected by *A*. *japonica* venom due to an intrinsic neutralizing activity in their hemolymph, indicating that these species must have acquired a neutralizer of *A*. *japonica* venom during evolution. These results give new insights into the characteristics of both the venom and LO components: *A*. *japonica* females have utilized the virus-like toxic venom factor to exploit a wider range of host species after the evolutionary process enabled them to use the LO factor for activation of the host hemolymph neutralizer precursor, although the non-habitual host *Drosophila* species possess an active intrinsic neutralizer in their hemolymph.

## Introduction

During evolution, endoparasitoid wasps have developed a variety of strategies for successful parasitism[1–3]. The innate immune system of host insects serves as a defense not only against microbial infection but also against parasitoids; therefore, habitual parasitoid wasps must manipulate the host immune system. In other words, only parasitoids that acquired the strategies to overcome the host defense systems have survived the long battle against host insects[4–8]. It is well known that many endoparasitoids harbor viruses or virus-like particles in their reproductive apparatus and that these particles are introduced into the host at parasitization[9–15]. Polydnaviruses (PDVs) are among the best-known examples of endoparasitoid symbiotic effectors[16–18]. PDVs manipulate the host defense system by using a variety of strategies, often categorized as passive or active[19–21]. As a passive strategy, PDV particles possess surface features that prevent the host from recognizing the parasitoid as non-self. For example, parasitoid *Cotesia kariyai* eggs and larvae are covered by many molecules of immunoevasive protein (IEP), one component of the *C*. *kariyai* PDV particles, which is not recognized as non-self by hemocytes of the wasp’s host[22–25]. An active strategy is suppression of the host immune system due to expression of various virulent factors coded on PDV genomes. Ankyrin-repeat motif proteins[26–28] and protein tyrosine phosphatases[29–31], which are widely distributed in many PDVs, have been reported as virulence factors that are responsible for disrupting the function of immune cells. Although the detailed mechanism of PDV-induced suppression of the host immunity is not fully understood, PDV-carrying parasitoid wasps are generally thought to owe successful parasitism to their PDVs.

Parasitoid wasps *A*. *japonica* are generalist parasitoids that successfully parasitize many *Drosophila* species but lack PDVs. Instead of PDVs, *A*. *japonica* wasps utilize a venom component for successful parasitization. We previously showed that the host *Drosophila* larvae are killed by envenomization at a dose that is naturally injected by *A*. *japonica* female wasps at parasitization[32, 33]. Such a toxic venom was demonstrated to be essential for the wasp to prevent host cellular immune defenses from killing the wasp’s progeny[33]. During natural parasitism, this toxicity is neutralized by subsequent injection of the lateral oviduct (LO) components; otherwise, the wasps cannot survive. Therefore, both the highly toxic venom and its neutralizer, LO, are indispensable for the successful parasitism of *A*. *japonica*. However, the functional consequences as well as the molecular natures of both factors remain ambiguous. Our present examination first revealed that the venom toxicity is due to a virus-ike entity because it was nullified by certain physical treatments such as UV irradiation and sonication, and precipitated by ultracentrifugation. Functional characterization of the LO components was then performed to elucidate how the LO factor neutralizes the venom toxicity. A series of characterizations demonstrated that the LO factor does not directly neutralize the venom toxicity but activates a neutralizer precursor that is present in the hemolymph of *D*. *melanogaster* larvae. Finally, a similar precursor of the venom neutralizer was found in the non-*Drosophila* insect *Spodoptera litura* larvae, which are also sensitive to *A*. *japonica* venom, while non-habitual host *Drosophila* species larvae retain the active form of the neutralizer that protects them from *A*. *japonica* parasitism.

## Materials and Methods

### Insects

*Drosophila melanogaster* have been maintained in our laboratory. *D*. *ficusphila* and *D*. *bipectinata* were collected in Iriomote-jima, Japan (Dr. Masahito Kimura (Hokkaido University) issued permission for collection) [32]. All fly strains were reared on cornmeal-malt-glucose-yeast medium at 23±1°C with a photoperiod of 15 h light: 9 h dark. *Asobara japonica* was collected in Tokyo, Japan, and reared in *D*. *simulans* or *D*. *melanogaster* as hosts. Parasitoids were maintained under the same temperature and light as the host strains[34].

*Spodoptera litura* was used for this study as an insect species example that is phylogenetically far from *Drosophila*. They were reared on an artificial diet (10% kidney beans, 10% wheat bran, 4.2% dried yeast, 0.5% ascorbic acid, 0.3% antiseptic reagents, and 1.3% agar (all w/w)) at 25°C with a photoperiod of 16 h light:8 h dark[35].

### Microinjection of venom with/without lateral oviduct fluid into test larvae

Venom reservoirs and lateral oviducts were dissected from *Asobara* wasp females and were separately placed into a drop of chilled phosphate-buffered saline (PBS: 8 mM Na_2_HPO_4_, 1.5 mM KH_2_PO_4_, 137 mM NaCl, and 2.7 mM KCl, pH 7.2). Venom reservoirs were lightly homogenized, lateral oviducts were squeezed with fine forceps, and centrifuged at 20,000 g for 15 min at 4°C to collect the supernatants[33]. In some experiments, the supernatant was further centrifuged at 120,000 g or 450,000 g for 30 min at 4°C.

Venom supernatant (after centrifugation at 20,000 g) (V) or a mixture of venom and lateral oviduct supernatants (V+LO) were diluted to 15 μl per a female with PBS. Six-day-old (third instar) *Drosophila* larvae were injected with 0.15 μl of each sample unless otherwise stated, put on the diet medium, and their survival rates were measured after injection. All test larva groups with survival rates higher than 60% at 24 h after injection normally produced puparia, and those with survival rates lower than 20% died within a few days. The fates of test larvae with survival rates between 20% and 60% at 24 h after injection varied in each case, and their subsequent survival rates are indicated in the figure legends.

When the above samples were injected into third instar larvae (body weight: 1.5 ± 0.1 mg) of *S*. *litura*, the amount of the sample was increased in proportion of *S*. *litura* larva weight to *Drosophila* larva weight.

### Preparation of Drosophila larval hemolymph

After washing *Drosophila* larvae well with PBS, the body was partly dipped in 50 μl of chilled PBS containing 0.1% *N* -phenylthiourea (PBS-PTU), and the hemolymph was collected by slightly tearing the cuticle using fine forceps on ice. The collected hemolymph was immediately centrifuged at 2,000 rpm for 3 min at 4°C, the protein concentration of the supernatant was measured by the method of Bradford using a Bio-Rad Protein assay reagent with bovine serum albumin as standard[36], and used for the the following experiments. Twenty larvae were usually necessary to collect about 10 μl hemolymph.

### Enzymatic treatment of venom components

After ultracentrifugation at 450,000 g for 30 min at 4°C, virus components (equivalent to two female) were treated by 0.01 μg/μl trypsin (Sigma-Aldrich, USA) at 25°C for 12 h, 0.2 units/μl DNase I (Takara, Japan) at 37°C for 2 h, 0.2 units/μl Benzonase (Merck, USA) at 37°C for 2 h, or 0.1 units/μl RNase A (Wako, Japan) at 37°C for 2 h. After each reaction, the reaction mixture was diluted 32 times and injected into test *Drosophila* larvae. Each ernzyme reaction mixture without virus components was used as a control sample.

### Measurement of protease activity using synthetic substrate-MCA

The release of 7-amino-4-methylcoumarin from synthetic peptide-MCA substrate (Boc-Ile-Glu-Gly-Arg-MCA (Peptide Institute, Inc., Japan)) during the hydrolase reaction was detected using a multimode detector DTX800 (Beckman Coulter) as described previously[33]; the hydrolase activity was regarded as a protease activity of each sample. Hemolymph collected from test *Drosophila* larvae was placed into a 50 μl drop of chilled PBS-PTU, immediately centrifuged at 300 g for 3 min at 4°C, and the supernatant was collected. Five μl of each hemolymph sample was incubated in 100 μl of 10 mM Tris-HCl buffer solution (pH 7.8) containing 20 μM peptide-MCA at 30°C for 1 h, and the increased fluorescence of the mixture was measured to calculate protease activities in the samples. One unit (U) of the protease activity was defined as that which hydrolyzed 1 nmol of the peptide-MCA substrate within 1 min.

### Production of anti-venom antibody

The pellet fraction after centrifugation at 450,000 g for 30 min was solubilized in 0.5% SDS and subcutaneously injected into rabbits with Freund’s complete adjuvant (TiterMax Gold, CytRx Corporation). Anti-venom IgG was precipitated by adding ammonium sulfate to 40% saturation and further purified by a protein A-Sepharose 4B column as described previously [37].

### Microscopic observation

For negative-staining electron microscopic analysis, carbon-coated 300-mesh copper grids were exposed to glow discharge in air for 20 s. Venom pellet suspensions (20 μl) were placed on grids and incubated for 3 min. Negative-staining was performed with 2% phosphotungstic acid (PTA, pH 7.2) for 30 sec[38]. The specimens were observed using a JEM-1400 transmission electron microscope (JEOL, Japan).

### Statistical analyses

For comparison of survival rates, body weights, and enzyme activities of test animals, Tukey’s HSD tests were carried out. A normality test, the Shapiro-Wilk test, showed that data sets do not deviate from the normality. All statistical analyses were performed using JMP 9.0.2 (SAS Institute).

## Results

### Functions of venom components

**Fig 1:**
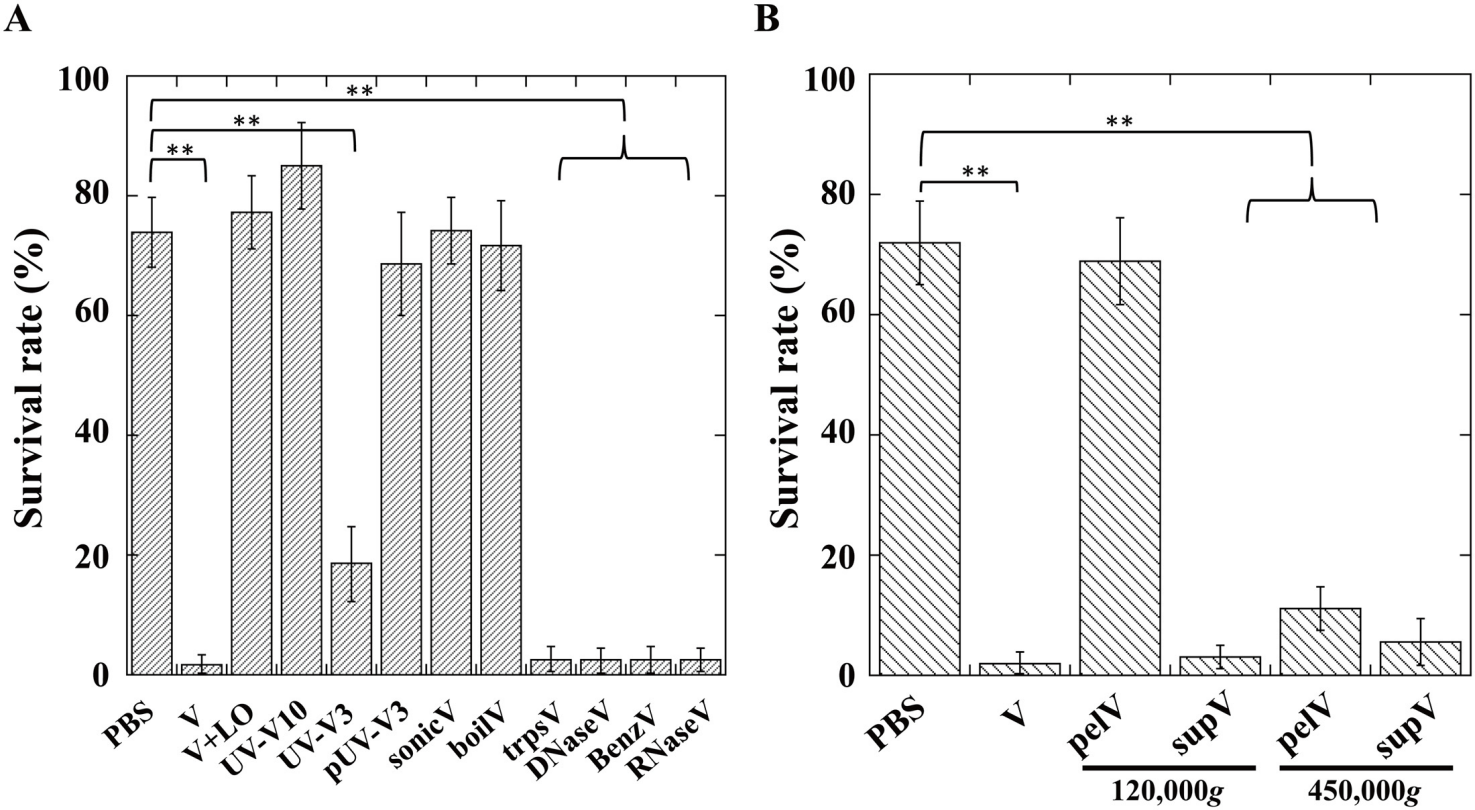
Survival rates of *Drosophila melanogaster* larvae one day after injection of indicated samples. (*A*) PBS, venom (V), venom plus lateral oviduct (V+LO), venom irradiated with UV for 10 min (UV-V10), venom irradiated with UV for 3 min (UV-V3), venom plus 10 μg/ml psoralen irradiated with UV for 3 min (pUV-V3), venom sonicated for 5 min (sonicV), venom boiled for 5 min (boilV), trypsinized venom (trpsV), DNase-treated venom (DNaseV), Benzonase-treated venom (BenzV), or RNase-treated venom (RNaseV) was injected. Each value represents the mean ± S.D. for six independent experiments performed separately. Significant differences are indicated by Tukey’s HSD (**P<0.01). (*B*) PBS, venom (V), pellet (pelV) or supernatant (supV) after centrifugation of venom at 120,000 g or 450,000 g was injected. Other explanations are as in (*A*).

**Fig 2:**
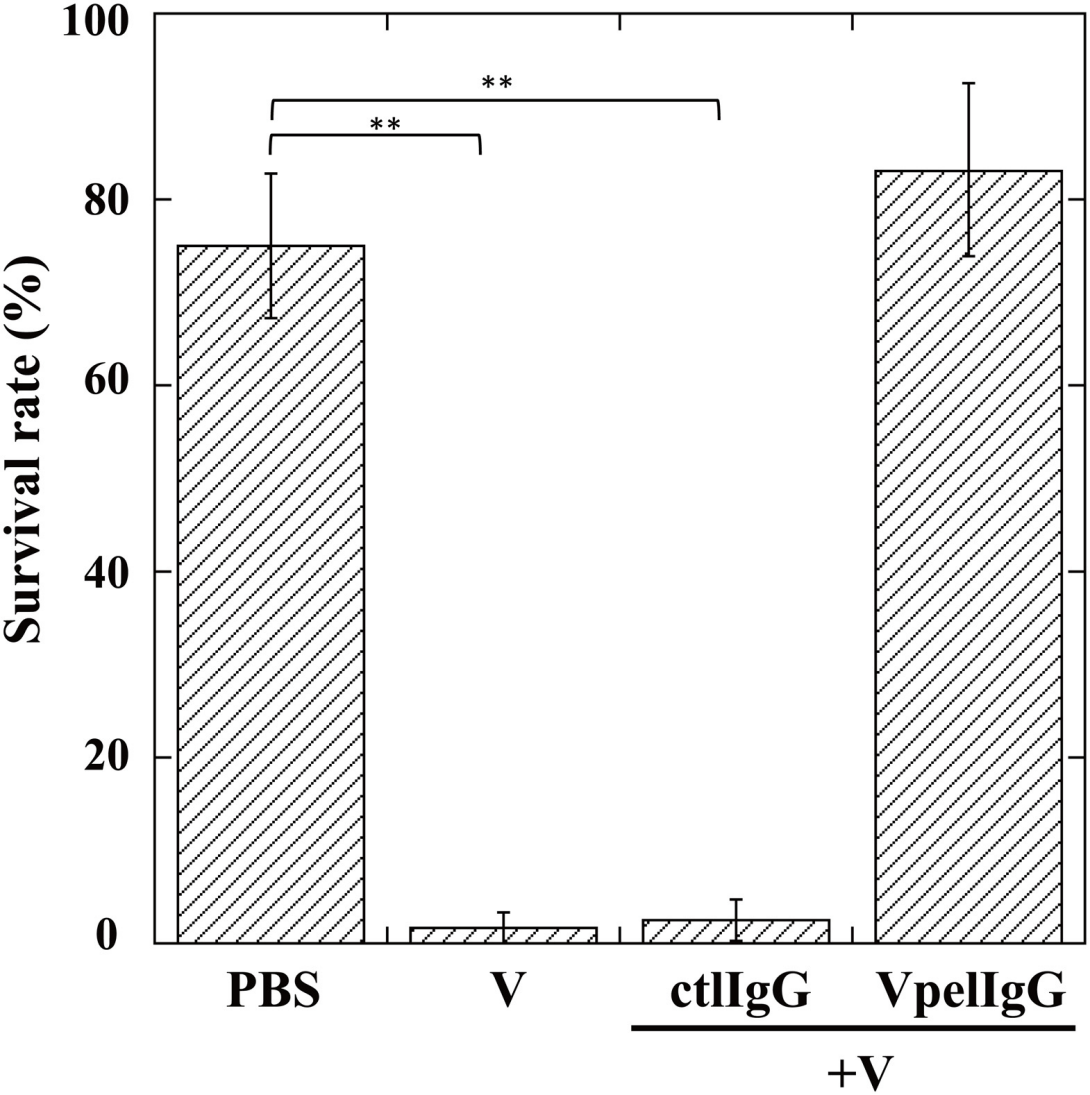
Survival rates of *Drosophila melanogaster* larvae one day after injection of PBS, venom (V), venom plus non-immunized IgG (V+ctlIgG), or venom plus anti-venom pellet (after centrifugation) IgG (V+VpelIgG). Venom components were treated with IgG for 3 h before injection into test *D*. *melanogaster* larvae. Each value represents the mean ± S.D. for five independent experiments performed separately. Significant differences are indicated by Tukey’s HSD (**P<0.01).

**Fig 3:**
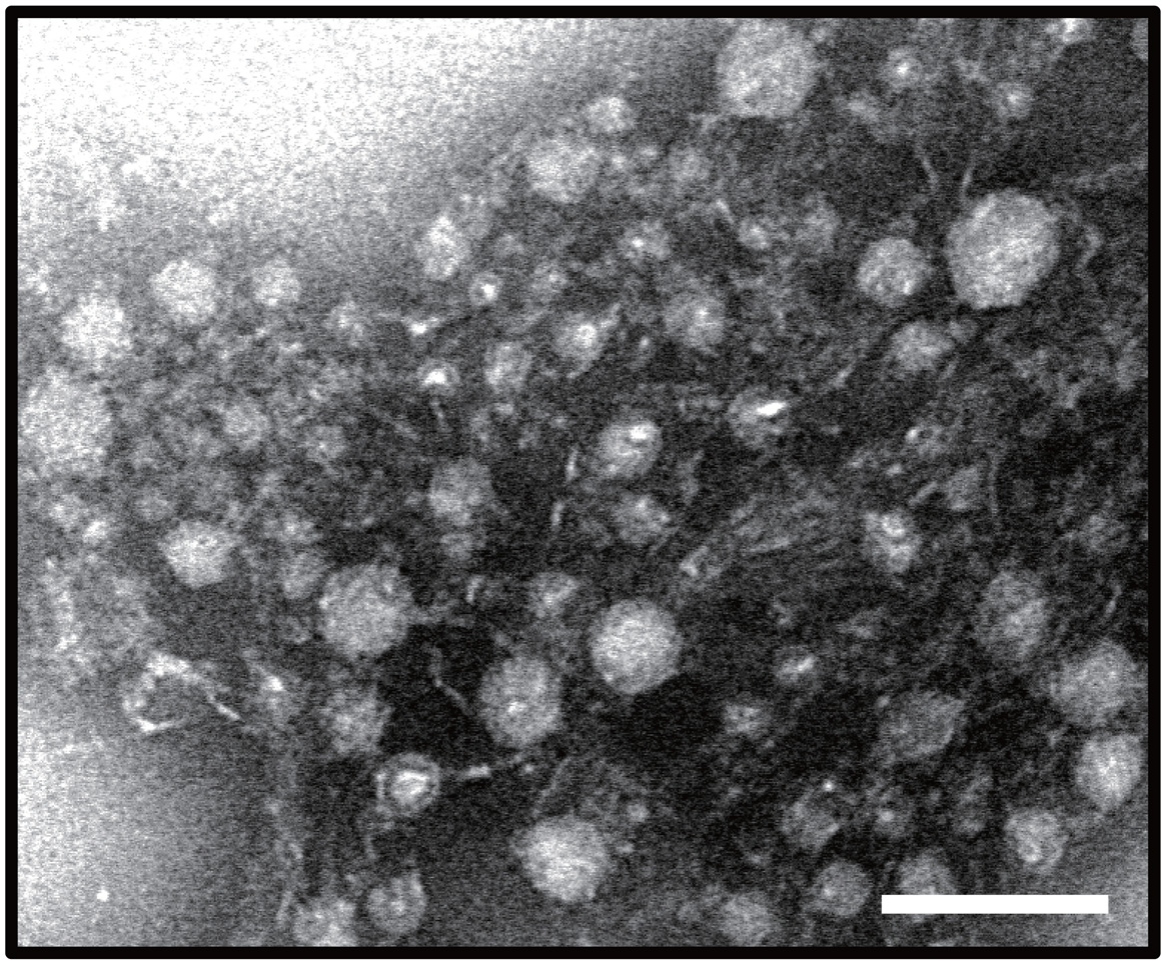
Transmission electron micrographs of ultracentrifuged precipitate of venom. *A*. *japonica* venom was centrifuged at 450,000 g for 1 h, and the pellet fraction was observed by transmission electron microscopy after negative-staining with aqueous phosphotungstic acid. Scale bar indicates 100 nm.

Introduction of only *A*. *japonica* venom components without subsequent injection of the oviduct components caused mortality of the host *Drosophila* larvae. To characterize the molecular nature of the venom toxic factor(s), we examined the effects of several physical or chemical treatments on the activity of the venom factor. Ultraviolet light (UV) irradiation, sonication, and boiling completely nullified the insecticidal effect of the venom factor. Although treatments with trypsin, DNase, Benzonase, or RNase did not affect its toxicity at all, addition of psoralen significantly shortened the period of UV irradiation required for inactivation of the venom toxicity, indicating the possibility that this toxicity is due to a virus-like particle with certain polynucleotides (Fig 1A), indicating the possibility that this toxicity is due to a virus-like particle containing certain polynucleotides. To assess this interpretation, venom components were centrifuged at 120,000 g or 450,000 g for 1 h. The toxic activity was not detected in the pellet after centrifugation at 120,000 g but was clearly detected in the pellet after centrifugation at 450,000 g (Fig 1B). These observations indicate that the venom toxic factor is not water-soluble but has a specific gravity high enough to be precipitated by ultracentrifugation at 450,000. Furthermore, the antibody against the pellet fraction after centrifugation at 450,000 g for 1 h neutralized the venom toxicity (Fig 2). To demonstrate the presence of certain virus particles in venom, the pellet fraction of the venom after centrifugation at 450,000 g was analyzed by electron microscopy. We found that the venom pellet fraction contained heterogeneous spherical virus-like particles whose diameters are approximately 20 to 40 nm (Fig 3), implying that virulent virus-like agent(s) could be one or some of these particles.

### Neutralizing effect of lateral oviduct components

**Fig 4:**
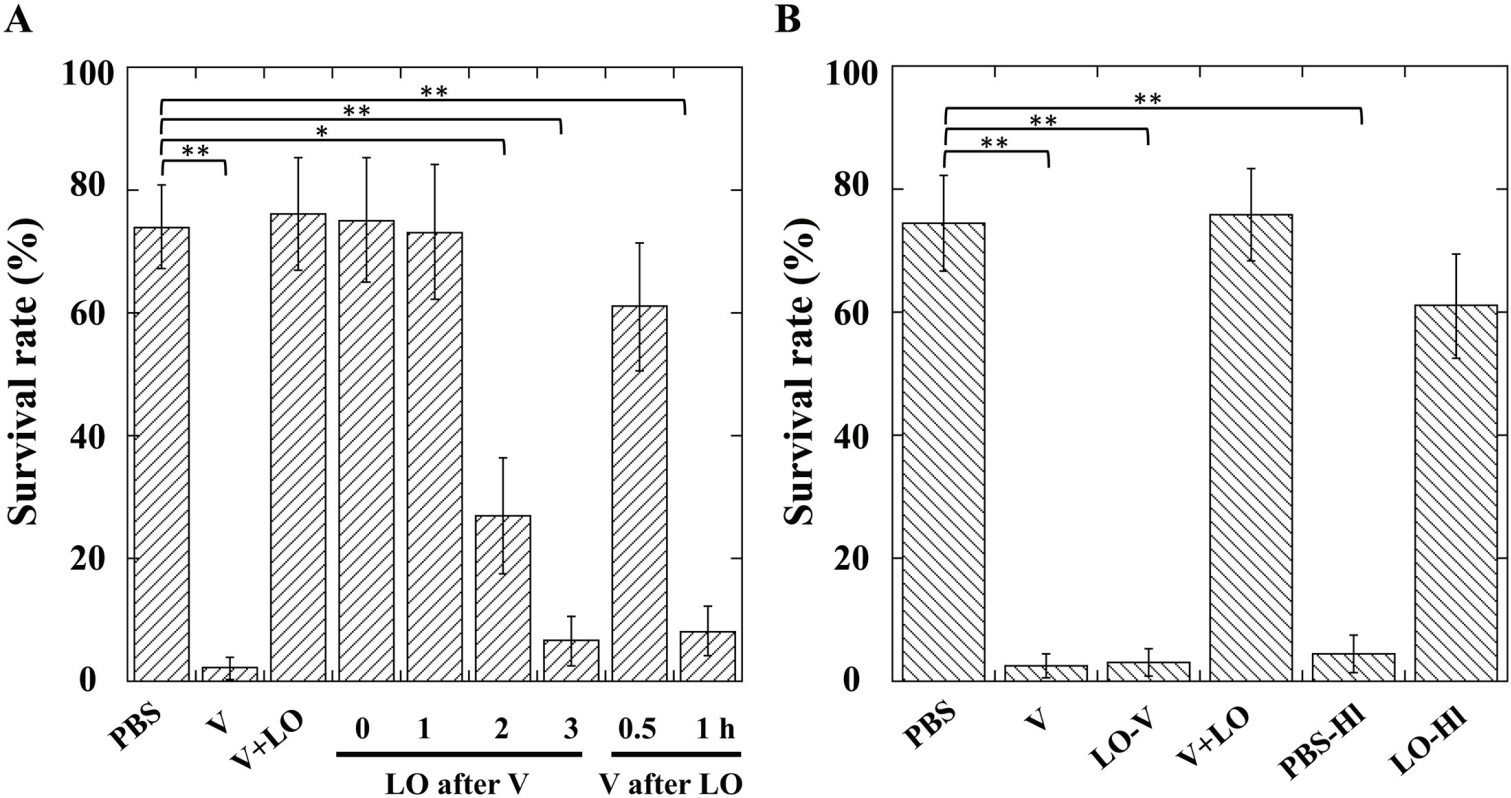
Survival rates of *Drosophila melanogaster* larvae one day after injection of indicated samples. (*A*) PBS, venom (V), or venom plus lateral oviduct (V+LO) was injected at indicated intervals before or after injection of venom. Survival rates of test larvae injected the LO factor 2 h after the venom injection: 12.6 ± 9.5% at 48 h, 8.5 ± 5.4% at 72 h, 0% at 96 h. Each value represents the mean ± S.D. for seven independent experiments performed separately. Significant differences are indicated by Tukey’s HSD (*P<0.05, **P<0.01). (*B*) PBS, venom (V), venom pretreated with lateral oviduct (LO-V), venom plus lateral oviduct (V+LO), or hemolymph of *D*. *melanogaster* larvae that had been pretreated with PBS (PBS-Hl) or lateral oviduct (LO-Hl) was injected. Each value represents the mean ± S.D. for six independent experiments performed separately. Significant differences are indicated by Tukey’s HSD (**P<0.01).

Although we previously showed that simultaneous injection of the LO components neutralizes the venom toxicity[32], we had not tested different timings of the LO component injection. We examined the effects of the LO injection at certain intervals before and after the venom injection. Injection of the LO components 1 h to 2 h prior to the venom injection showed a significant neutralizing effect on the venom toxin, although injection of the venom 3 h before did not. Injection of the LO components 0.5 h after the venom injection also neutralized the venom, while injection 1 h later did not (Fig 4A). Furthermore, direct pretreatment of venom factors with the LO components before injection did not neutralize the venom toxicity when the LO factor was removed by ultracentrifugation (Fig 4B). The hemolymph itself prepared from larvae preinjected with the LO components showed a neutralizing effect similar to the direct injection of the LO components (Fig 4B), suggesting that the LO components did not inactivate the toxic venom directly but activated a precursor form of the hemolymph neutralizer.

### Effects of *A*. *japonica* venom and oviduct components on *Spodoptera litura*

**Fig 5:**
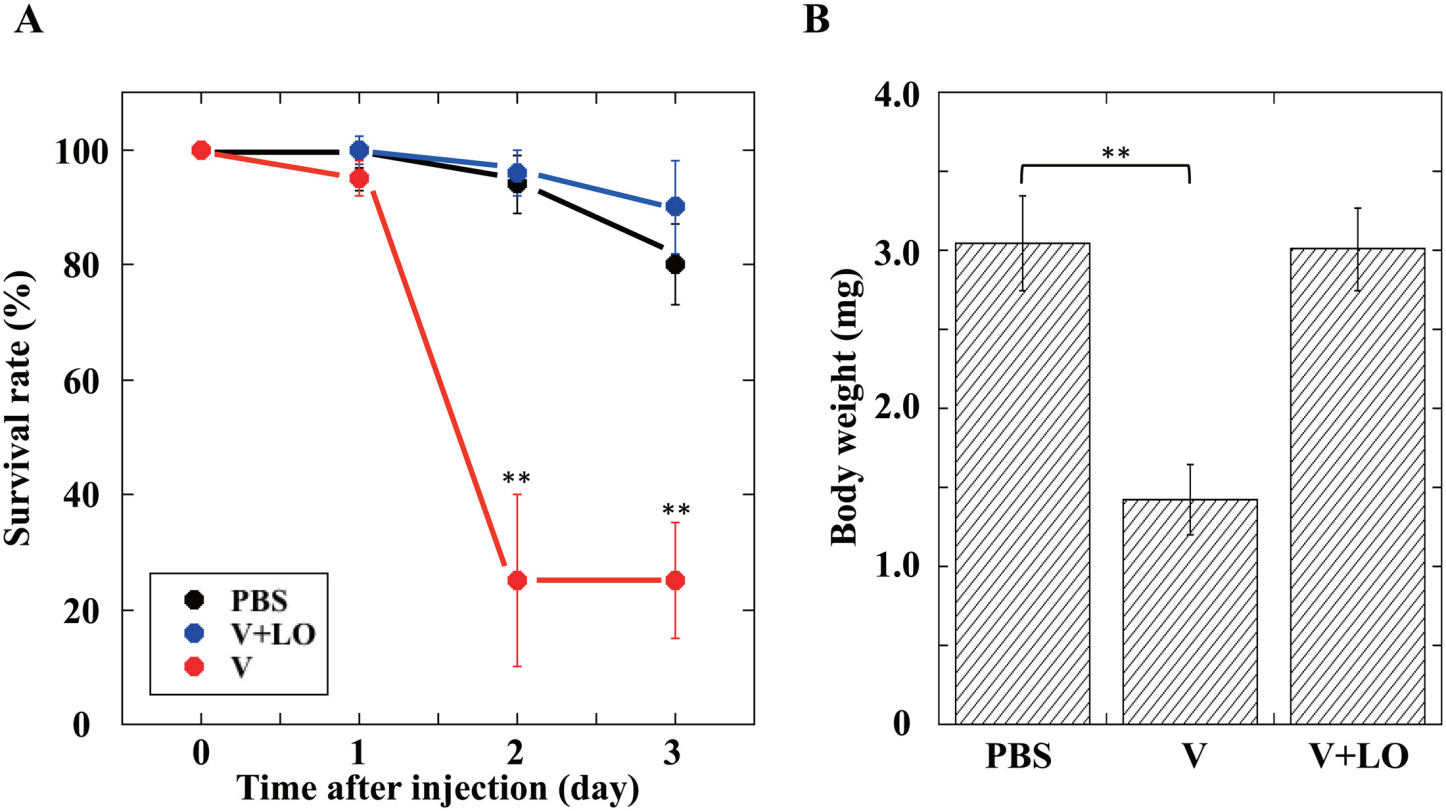
Effects of *A*. *japonica* venom and LO components on survival rates and growth of *Spodoptera litura* larvae. (*A*) Life span curves of *S*. *litura* larvae after injection of PBS, venom (V), or venom plus lateral oviduct (V+LO). The amount injected was increased with the increasing ratio of *S*. *litura* larva weight to *Drosophila* larva weight. Third instar larvae of *S*. *litura* (body weight: 1.50 ± 0.10 mg) were used for this experiment. Each value represents the mean ± S.D. for ten independent experiments performed separately. Significant differences compared to the value at 0 day are indicated by Tukey’s HSD (**P<0.01). (*B*) Body weights of surviving *S*. *litura* larvae one day after injection of PBS, venom (V), or venom plus lateral oviduct (V+LO). Other explanations are as in (*A*).

The biological effects of *A*. *japonica* venom and oviduct components have been examined mainly using *D*. *melanogaster* larvae as the habitual host but not using the non-habitual hosts or non-*Drosophila* insect species[33]. We examined the effects of these two components on larvae of the common cutworm *Spodoptera litura*. Injection of only *A*. *japonica* venom without the LO components caused approximately 75% mortality in *S*. *litura* larvae (Fig 5A). Moreover, significant growth retardation of surviving *S*. *litura* larvae was observed one day after the venom injection (Fig 5B). However, *A*. *japonica* venom did not have any detrimental effects on *S*. *litura* larvae when it was co-injected with the LO components, indicating that the effects of both components, venom and LO factors, affected a non-*Drosophila* insect species in a way similar to the habitual host species, *D*. *melanogaster*.

### Effects of *A*. *japonica* venom on *D*. *bipectinata* and *D*. *ficusphila*

**Fig 6:**
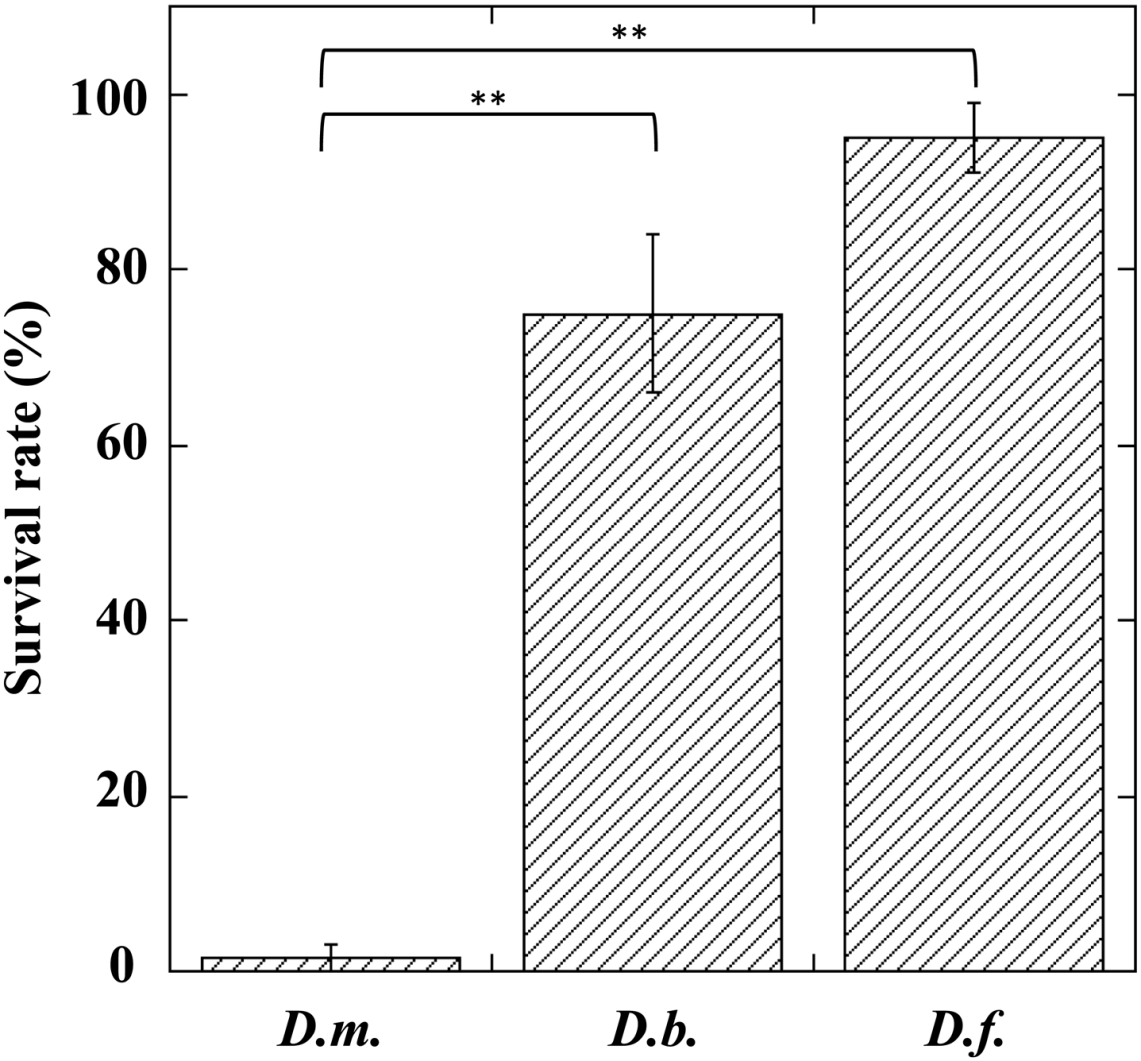
Survival rates of *Drosophila melanogaster* (*D*.*m*.), *Drosophila bipectinata* (*D*.*b*.), and *Drosophila ficusphila* (*D*.*f*.) larvae one day after injection of *A*. *japonica* venom. Each value represents the mean ± S.D. for eight independent experiments performed separately. Significant differences are indicated by Tukey’s HSD (**P<0.01).

**Fig 7:**
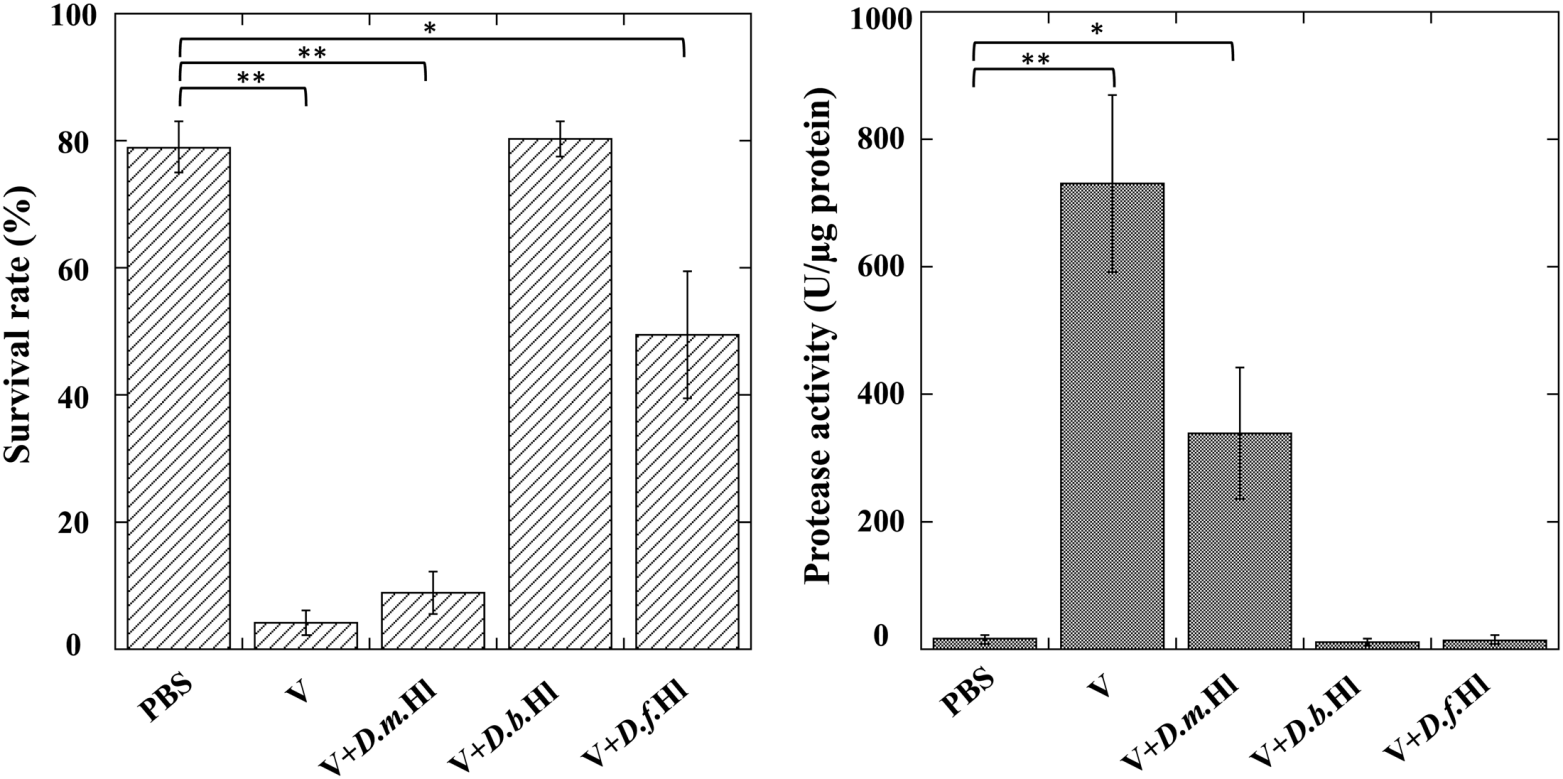
Survival rates (*A*) and hemolymph protease activities (*B*) of *Drosophila melanogaster* one day after injection of indicated samples. PBS, venom (V), or venom plus larval hemolymph of *D*. *melanogaster* (V+*D*.*m*.Hl), *D*. *bipectinata* (V+*D*.*b*.Hl), or *D*. *ficusphila* (V+*D*.*f*.Hl). Survival rates of test larvae injected the venom factor together with *D*. *ficusphila* hemolymph: 49.3 ± 10.6% at 48 h, 48.6 ± 11.1% at 72 h, 46.9 ± 11.5% at 96 h. Each value represents the mean ± S.D. for eight independent experiments performed separately. Significant differences are indicated by Tukey’s HSD (*P<0.05, **P<0.01).

We previously reported that the injection of *A*. *japonica* venom did not have a toxic effect on the non-habitual host species of *Drosophila*, *D*. *ficusphila*[33]. To confirm and expand this observation, we performed the same experiments using another non-habitual *Drosophila* host species, *D*. *bipectinata*. As we expected, *A*. *japonica* venom did not have any toxic effect on *D*. *bipectinata* (Fig 6), thus implying that these non-habitual *Drosophila* host larvae possess an active neutralizer in the hemolymph. To assess this interpretation, we injected *A*. *japonica* venom together with hemolymph prepared from *D*. *bipectinata* or *D*. *ficusphila* larvae into *D*. *melanogaster* larvae. Although the hemolymph of *D*. *melanogaster* larvae did not show any neutralizing activity at all, both hemolymphs prepared from *D*. *bipectinata* and *D*. *ficusphila* larvae significantly neutralized *A*. *japonica* venom toxicity (Fig 7A).

We previously found that the proteolytic activity in the plasma (void of hemocytes) was drastically elevated following the venom injection, but its elevation was blocked by coinjection of the LO components[33]. Because this enzymatic elevation is closely related to the successful parasitism, measurement of this enzyme activity is useful to evaluate the effectiveness of the venom activity as well as the neutralizer activity. In order to examine the effects of *D*. *bipectinata* and *D*. *ficusphila* larval hemolymph on the venom-induced proteolytic activity, each plasma fraction prepared from these larvae was coinjected with the venom components. *D*. *melanogaster* larval hemolymph did not affect the proteolytic activity at all, while the plasma of both *D*. *bipectinata* and *D*. *ficusphila* larvae significantly blocked the venom-induced elevation of proteolytic activity (Fig 7B). These results indicate that these two non-habitual *Drosophila* host species, *D*. *ficusphila* and *D*. *bipectinata*, possess the hemolymph factor(s) that neutralize the toxic venom factor of *A*. *japonica* to repress the venom-induced elevation of the proteolytic activity.

## Discussion

The parasitic success of many parasitoid wasps inside their hosts is attributed in large part to symbiotic viruses or virus-like particles (VLPs). In the absence of these factors, the eggs are recognized as a dangerous foreign substance and destroyed by the host defense system. The most studied symbionts are the polydnaviruses (PDVs), which have been investigated for more than 40 years[18]. In this study, we focused on *A*. *japonica* to analyze the parasitism strategy of a parasitoid wasp lacking PDVs. Instead of PDVs, *A*. *japonica* females use their toxic venom to prevent the host defense system from killing their progeny. Prior studies demonstrated that *A*. *japonica* venom especially diminished the host cellular defense reactions, spreading and phagocytosis, by host *D*. *melanogaster* hemocytes[33]. Moreover, the venom significantly induced cell death in *D*. *melanogaster* hemocytes by coincubation for one hour. Although a highly toxic venom is essential for successful parasitization by *A*. *japonica* wasps, the venom toxicity must be neutralized soon after the manipulation of the host defense system; otherwise, the wasps would lose their hosts by killing them. For this neutralization, *A*. *japonica* females use the proteinaceous factor(s) in the lateral oviduct (LO) that are introduced with the eggs a few seconds after venomization. The present study showed that the LO factor can neutralize the venom toxin by its injection into the host hemocoel pre- or post-venomization: LO-factor injection can function from two hours before venomization to half an hour after venomization. Furthermore, the hemolymph itself prepared from *D*. *melanogaster* larvae preinjected with the LO factor can neutralize the venom toxin, suggesting that the LO factor does not directly affect the venom components but activates a precursor of the neutralizer in *D*. *melanogaster* hemolymph. This interpretation was supported by the fact that the precipitate after the ultracentrifugation of the venom pretreated with the LO components retained high toxicity.

Interestingly, hemolymph of both *D*. *bipectinata* and *D*. *ficusphila* larvae, which are non-habitual *Drosophila* host species of *A*. *japonica*, was able to neutralize *A*. *japonica* venom without pretreatment by *A*. *japonica* LO factor, indicating that these species larvae possess an intrinsic hemolymph factor to neutralize the toxic venom. In contrast, the venom effect on the non-*Drosophila* insect species *Spodoptera litura* larvae was the same as that on habitual host *D*. *melanogaster*: *S*. *litura* larvae were detrimentally affected by injection of *A*. *japonica* venom but coinjection of the LO factor neutralized the venom. Based on these results, it is reasonable to presume that interaction between *A*. *japonica* (or the ancestral parasitoid species) and *Drosophila* species must have continued for a long time, and during that long period, some flies, such as *D*. *bipectinata* and *D*. *ficusphila*, acquired the active hemolymph neutralizer that inactivates *A*. *japonica* venom. This inference is partly supported by the fact that the generalist endoparasitoid *A*. *japonica* succeeded in parasitizing 12 of a total 14 tested *Drosophila* species, and that *A*. *japonica* failed to parasitize only two species, *D*. *bipectinata* and *D*. *ficusphila*[39]. In other words, because *A*. *japonica* females have acquired the highly toxic venom factor to overcome various immune reactions of *Drosophila* species, they have become generalist parasitoids in the evolutionary process, although a few species that have acquired the active neutralizer against the venom succeed in escaping from parasitism by *A*. *japonica*.

In the previous study[33], we also found that the proteolytic (peptide MCA substrate hydrolase) activity of the host *D*. *melanogaster* hemolymph was drastically elevated by injection of *A*. *japonica* venom when we used the MCA substrate (Boc-Ile-Glu-Gly-Arg-MCA) that is generally utilized as a Factor Xa substrate[40]. This elevation of proteolytic activity seems to be closely related to the insecticidal effect of the venom because coinjection of the venom with the LO components blocked elevation of the enzymatic activity. We demonstrated that both *D*. *bipectinata* and *D*. *ficusphila* larval hemolymphs also blocked the elevation of the venom-induced proteolytic activity without pre-injection of the *A*. *japonica* LO components. Therefore, it is reasonable to assume that *D*. *bipectinata* and *D*. *ficusphila* larval hemolymph intrinsically possess an active inhibitory factor that suppresses the venom-induced elevation of the proteolytic activity, although *D*. *melanogaster* larvae can induce the active inhibitory hemolymph factor only with the aid of the LO factor.

In this study, we provided chemical, physical, and morphological evidence that the *A*. *japonica* toxic venom factor could be a virus-like entity. The venom toxicity was significantly impaired by UV irradiation, sonication, and boiling but not by treatment with trypsin. The toxic venom compound was also precipitated by ultracentrifugation at 450,000 g for one hour. Furthermore, the precipitate was shown to contain spherical virus-like particles by electron microscopic analysis. Although the particles are heterogeneous in size, most of them are small spheres approximately 20 to 40 nm in diameter. Therefore, if these small sphere particles are the venom toxic factors, they are completely different from the larger size viruses reported in parasitoid wasps, which include PDVs[17, 18, 41], entomopoxviruses[42, 43], and ascoviruses[9, 44–47]. Nevertheless, if we postulate that the *A*. *japonica* venom factor belongs to an already known insect virus family, the candidate virus group must be limited to small RNA viruses such as picornavirus [48–50] or small DNA viruses such as parvovirus [51–53].

Because both small RNA and DNA viruses have been reported as pathogenic viruses in a broad range of insect species, these viruses could be regarded as candidates for the *A*. *japonica* toxic venom factor. We have tried several times to identify a virus genomic nucleotide sequence by using Next-generation sequencing technologies, but all trials failed, mostly due to significant contamination by a large amount of *Wolbachia* in the venom preparation after ultracentrifugation. Further purification of the venom virus preparation is necessary for successful sequencing.
